# Removal of unwanted variation reveals novel patterns of gene expression linked to sleep homeostasis in murine cortex

**DOI:** 10.1186/s12864-016-3065-8

**Published:** 2016-10-25

**Authors:** Jason R. Gerstner, John N. Koberstein, Adam J. Watson, Nikolai Zapero, Davide Risso, Terence P. Speed, Marcos G. Frank, Lucia Peixoto

**Affiliations:** 1 Washington State University, Elson S. Floyd College of Medicine, Spokane, WA 99202 USA; 2 Department of Neuroscience, Perelman School of Medicine University of Pennsylvania, Philadelphia, PA 19104 USA; 3 Division of Biostatistics, School of Public Health, University of California, Berkeley, CA 94720 USA; 4 Department of Statistics, University of California, Berkeley, CA USA; 5 Department of Mathematics and Statistics, The University of Melbourne, Melbourne, VIC Australia; 6 Bioinformatics Division, The Walter and Eliza Hall Institute of Medical Research, Parkville, VIC Australia

**Keywords:** Sleep, Sleep deprivation, Circadian, Microarray, Gene, mRNA, Transcriptomics

## Abstract

**Background:**

Why we sleep is still one of the most perplexing mysteries in biology. Strong evidence indicates that sleep is necessary for normal brain function and that sleep need is a tightly regulated process. Surprisingly, molecular mechanisms that determine sleep need are incompletely described. Moreover, very little is known about transcriptional changes that specifically accompany the accumulation and discharge of sleep need. Several studies have characterized differential gene expression changes following sleep deprivation. Much less is known, however, about changes in gene expression during the compensatory response to sleep deprivation (i.e. recovery sleep).

**Results:**

In this study we present a comprehensive analysis of the effects of sleep deprivation and subsequent recovery sleep on gene expression in the mouse cortex. We used a non-traditional analytical method for normalization of genome-wide gene expression data, Removal of Unwanted Variation (RUV). RUV improves detection of differential gene expression following sleep deprivation. We also show that RUV normalization is crucial to the discovery of differentially expressed genes associated with recovery sleep. Our analysis indicates that the majority of transcripts upregulated by sleep deprivation require 6 h of recovery sleep to return to baseline levels, while the majority of downregulated transcripts return to baseline levels within 1–3 h. We also find that transcripts that change rapidly during recovery (i.e. within 3 h) do so on average with a time constant that is similar to the time constant for the discharge of sleep need.

**Conclusions:**

We demonstrate that proper data normalization is essential to identify changes in gene expression that are specifically linked to sleep deprivation and recovery sleep. Our results provide the first evidence that recovery sleep is comprised of two waves of transcriptional regulation that occur at different times and affect functionally distinct classes of genes.

**Electronic supplementary material:**

The online version of this article (doi:10.1186/s12864-016-3065-8) contains supplementary material, which is available to authorized users.

## Background

Sleep is thought to be controlled by two processes, 1) a homeostatic process that determines sleep need (or pressure), and 2) a circadian process that determines the timing of sleep and wakefulness. A robust index for sleep need is known as delta power, which refers to “delta” (1–4 Hz) oscillations in the electroencephalogram (EEG) of non-rapid eye movement (NREM) sleep. Delta power increases with increased sleep pressure, and declines following sleep. Therefore, sleep deprivation increases delta power, which then naturally decreases during recovery sleep. Previous studies have shown that EEG delta power is under genetic control [1], suggesting that specific genes contribute to sleep homeostasis. Nevertheless, the molecular mechanisms that regulate sleep need remain incompletely described.

Genome-wide technologies have been used to interrogate gene expression changes that follow sleep deprivation in the mouse brain [2, 3], but there is little agreement between studies. There are also no genome-wide studies that characterize transcriptional changes that occur during subsequent recovery sleep. A major challenge when analyzing genome-wide data in the brain in response to behavior is isolating the signal of interest from other factors (batch effects) that simultaneously influence gene expression. We have previously shown that batch effects are widespread in genome-wide studies of gene expression in experimental neuroscience [4]. Several data normalization strategies are available to correct these batch effects, including Removal of Unwanted Variation (RUV). RUV adjusts for batch effects by performing factor analysis on control genes or replicate samples [5–7]. We have also shown that RUV normalization leads to increased power and reproducibility of results [4]. We now employ RUV to generate an integrated cross-laboratory analysis of differential gene expression following sleep deprivation in the mouse brain. We also provide the first comprehensive genome-wide assessment of transcripts from mouse cortex during recovery sleep. Our analysis improves detection of differentially expressed genes following sleep deprivation and shows that recovery sleep reverses the transcriptional changes it causes. This latter process occurs in waves that happen at different times during recovery sleep and affect functionally distinct classes of genes.

## Results

**Fig. 1:**
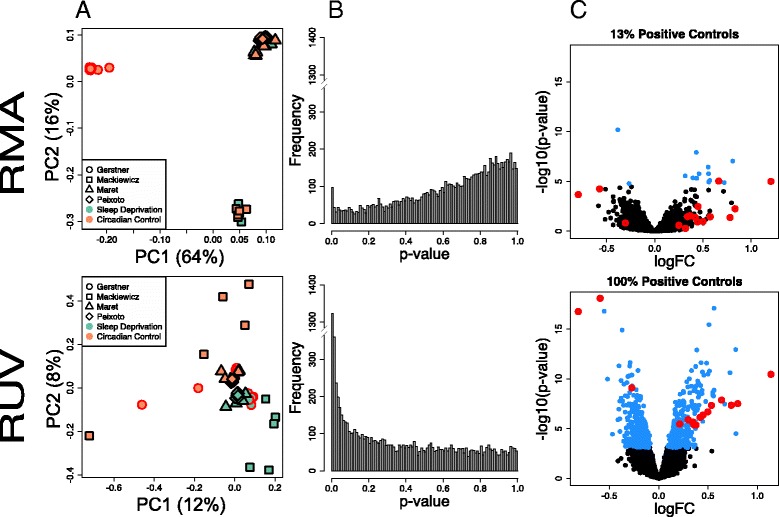
Integrated analysis of the effect of 6 h of sleep deprivation in the murine cortex. **a** Scatterplots of first two principal components (PC, log-scaled, centered intensities) following RMA and RUV normalization. Percent variance explained by each PC in parenthesis. Triangles denote samples from the Maret et al. 2007 study [2] (whole brain), square samples from the Mackiewicz et al. 2007 study [3] (cortex), rhombus the samples from Vecsey, Peixoto et al. 2012 [8] (hippocampus), and circles are samples from this study (cortex). In green, samples following 6 h of sleep deprivation (SD); in orange, time of day matched controls (CC). Samples cluster according to array platform (PC1) and lab (PC2) following Quantile normalization. After applying RUV, samples cluster according to treatment (PC2). **b** Distribution of unadjusted *p*-values for tests of differential expression between SD and CC samples following Quantile and RUV normalization. The distribution of *p*-values following Quantile normalization is not uniform and biased towards 1. RUV returns uniformity to the *p*-value distribution and increases discovery of differentially expressed genes (genes that have a low *p*-value). **c** Volcano plot of differential expression (−log10 *p*-value vs log fold change) of Quantile and RUV normalized samples. Genes with an FDR <0.01 are highlighted in blue. Positive controls are circled in red; RUV increases the detection of known differentially expressed genes from 0 to 100 %. PCA plots were performed using the R/Bioconductor package EDASeq (v. 2.0.0). RUVs normalization was performed using the R/Bioconductor package RUVSeq (v. 1.0.0). Differential expression analysis was performed using R/Bioconductor package limma

We first wanted to obtain a reliable estimate of the differential gene expression changes caused by sleep deprivation. To do this, we subjected mice to 5–6 h of sleep deprivation and different amounts of recovery sleep. Control mice (left undisturbed in home cages) were sacrificed at matching circadian times and cortical mRNA was isolated from experimental and control groups for microarray analysis (see methods for more details). We then integrated our data with that from three other studies performed in mouse cortex, whole brain and hippocampus [2, 3, 8]. We began with a traditional method of normalization, Robust multi-array average (RMA) plus quantile normalization [9], to integrate the data. The resulting principal component (PC) analysis showed that 64 % of the variability was due to platform (PC 1) while 16 % was due to different laboratories (PC 2; Fig. 1a). These results show that traditional normalization methods are not well suited to integrate data across laboratories or array platforms. Following RUV normalization, however, samples group according to treatment (sleep deprivation versus controls) and not laboratories, platform (Fig. 1a) or tissue type. We then evaluated the impact of normalization method in differential expression analysis. RUV restored the *p*-value histogram of differential expression to its expected distribution (Fig. 1b), increased detection of differentially expressed genes, and recovered 100 % of positive control genes known to respond to sleep deprivation (Fig. 1c). Positive control genes used in our study (see Methods) are listed in Additional file 1. The list of differentially expressed genes in response to sleep deprivation regardless of tissue for the integrated study is available in Additional file 2. Our results show that a method as effective as RUV normalization is required for the proper characterization of differentially expressed genes across labs and platforms in meta-analysis studies, as we have shown for RNA-seq data [4].

**Fig. 2:**
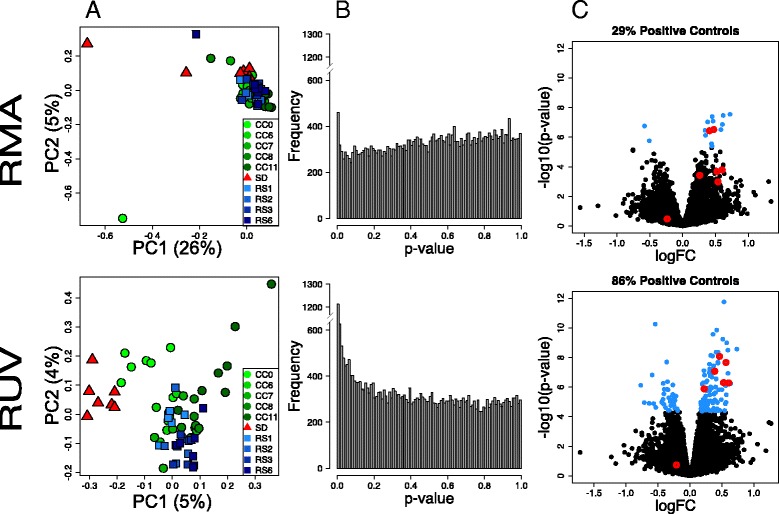
Analysis of the effect of sleep deprivation and subsequent recovery sleep in the murine cortex. **a** Scatterplots of first two principal components (log-scaled, centered counts) following RMA and RUV normalization. Percent variance explained by each PC in parenthesis. Triangles denote samples collected following sleep deprivation (SD), circles control samples matched by time of day (CC, number indicates ZT time) and squares samples collected after recovery sleep (RS). Samples only cluster according to treatment after RUV normalization. **b** Distribution of unadjusted *p*-values for tests of differential expression between one hour of recovery sleep (RS1) and control samples following RMA and RUV normalization. RUV increases discovery of differentially expressed genes (genes that have a low *p*-value). **c** Volcano plot of differential expression (−log10 *p*-value vs log fold change) of RMA and RUV normalized samples. Genes with an FDR <0.01 are highlighted in blue. Positive controls are circled in red; RUV increases the detection of known differentially expressed genes following recovery sleep. PCA plots were performed using the R/Bioconductor package EDASeq (v. 2.0.0). RUVs normalization was performed using the R/Bioconductor package RUVSeq (v. 1.0.0). Differential expression analysis was performed using R/Bioconductor package limma. The analysis was based only on samples were collected from current laboratory

We were also interested in determining the effects of recovery sleep on sleep deprivation-induced gene expression, as this has been minimally explored. Separate groups of mice were sleep-deprived and allowed to recover for 1, 2, 3, and 6 h (RS1, RS2, RS3, and RS6, respectively) to identify changes in genome-wide expression during recovery sleep. We again compared the effect of using traditional normalization (RMA) versus RUV on the characterization of differential gene expression after sleep deprivation or varying lengths of recovery sleep. The PC scatterplots revealed a lack of grouping of biological replicates with RMA, indicative of the presence of confounding factors or “batch effects” in the data (Fig. 2a). Unlike our previous analysis (Fig. 1), no obvious grouping attributable to technical factors was found. Following RUV normalization, the PCA plots showed the expected segregation of groups according to treatment (Fig. 2a). The effect of data normalization is also evident in the analysis of differential expression. RUV analysis improved the detection of differentially expressed genes following one hour of recovery sleep (RS1, Fig. 2b), and recovered 86 % of the positive controls (Fig. 2). Improvement was also seen at all other time-points (RS2, RS3 and RS6, Additional file 3). These results show that RUV normalization, or similar methodology geared to address batch effects, is necessary for accurate detection of differential expression not only across but also within studies. Genes differentially expressed at all time points in this study relative to controls can be found in Additional file 4. It is also known that time of day by itself can profoundly impact brain gene expression in the brain. To address the possible confound of circadian time in the fold changes observed following RS, we compared three controls samples to each other, CC0, CC6 and CC11, which correspond to circadian time ZT = 0, ZT = 6 and ZT = 11 respectively. The results are available in Additional file 5 and were taken into account on the interpretation of gene expression changes brought on by RS.

**Fig. 3:**
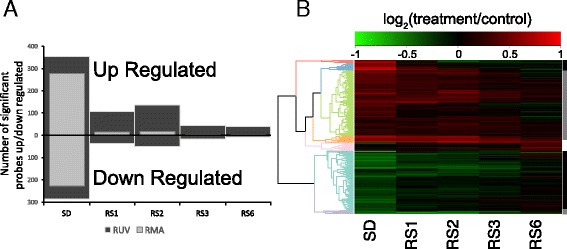
The effect of data normalization in differential expression following recovery sleep. **a** Bar graph displaying the number of up and downregulated probesets detected at each time point relative to time-of-day matched controls following RMA (*light grey*) or RUV (*dark grey*) normalization. RUV normalization profoundly affects the detection of differentially expressed genes following various lengths of recovery sleep. Positive controls for genes differentially expressed following 1 h of recovery sleep after were obtained as detailed in Methods. **b** Heatmap of differentially expressed probesets detected using RUV normalization relative to circadian time-matched controls. In red, upregulated genes. In green, downregulated genes. Clustering based on patterns of gene expression is represented by the dendrogram and color coded. Genes responding within 1–3 h to recovery sleep are indicated by black bars (fast responders), while genes that respond at 6 h are indicated by grey bars (slow responders, *grey bar*). Genes upregulated by sleep deprivation show two different patterns of response within the first three hours (red and pink clusters, *black bar*) and two different patterns of recovery at 6 h (green and orange clusters, *grey bar*). The majority of genes upregulated by sleep deprivation respond slowly with recovery sleep. The majority of genes downregulated are fast responders (mint green, *black bar*), while a very small proportion recovers within 6 h (lilac, *grey bar*). SD, sleep deprivation; RS1, sleep deprivation followed by 1 h of recovery sleep; RS2, sleep deprivation followed by 2 h of recovery sleep; RS3, sleep deprivation followed by 3 h of recovery sleep; RS6, sleep deprivation followed by 6 h of recovery sleep

The discharge of sleep need in mammals is a gradual process, including events that normalize within the first 3 h (e.g. NREM EEG delta power) and others that may take 6–12 h to appear (e.g. changes in sleep time) [1, 10]. We therefore plotted the number of significant probesets altered by sleep deprivation and recovery sleep relative to their respective circadian time-point controls following both RMA and RUV normalization (Fig. 3a). Compared to RMA, RUV analysis greatly improved the detection of significant genes for all groups examined. Its impact is more pronounced on the detection of differential expression during recovery sleep. Overall, the number of genes upregulated outnumbered those that are downregulated at all time-points.

**Fig. 4:**
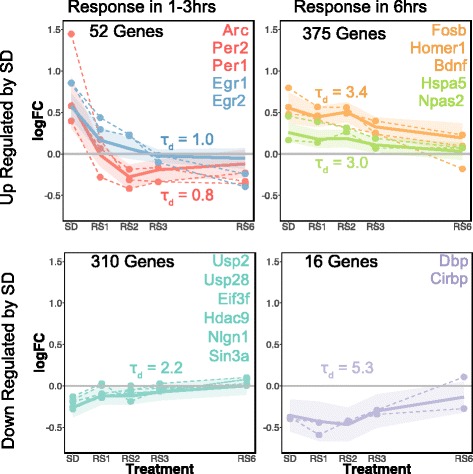
Patterns of gene expression regulation during recovery sleep. Plots of log-fold change (logFC) of differential expression relative to controls versus time since sleep deprivation. Color coding corresponds to clusters on Fig. 3b. Plots for genes representative of different expression patterns are shown as dashed lines. The ordering of the gene names within genes of the same cluster (*same color*) reflects the ordering of the plots. The average and standard deviation for each cluster is shown as solid lines and shaded area. Patterns of expression are divided in two classes: ‘fast’ responders (genes that reach basal values within to 1–3 h of RS) and ‘slow’ responders (genes that reach basal values >3 h). Time constants for the change during recovery sleep for average of each cluster are shown

We then generated a heatmap of differentially expressed genes detected using RUV normalization relative to circadian time-matched controls (Fig. 3b). The heatmap and subsequent clustering of gene expression across time-points revealed different patterns of gene expression relative to the amount of recovery sleep. We identified seven clusters of genes based on their expression pattern across recovery sleep (Additional file 6). These seven clusters can be grouped into three categories: genes that recover within 1–3 h (“fast responders”), genes that recover by 6 h of recovery sleep (“slow responders”), and genes unaffected by sleep deprivation, but upregulated by the 6^th^ hour of recovery. Examples of genes belonging to each category can be found in Fig. 4 and Additional file 6.

The majority of genes upregulated by sleep deprivation were slow responders, while the majority of genes downregulated by sleep deprivation were fast responders (the exception is a small cluster of 16 genes that includes *Cirbp* and *Dbp*), supporting the pattern observed in Fig. 3a. Interestingly, a more fine-grained analysis of these results showed that transcripts known to be upregulated by sleep deprivation (such as *Arc* and *Homer1*) [2, 3] include both ‘slow’ and ‘fast’ responders. Transcripts that recover by 1–3 h included *Arc*, *Per1*, *Per2*, *Egr1*, and *Egr2*, while transcripts that recovered by 6 h included *Homer1*, *BDNF*, *Fosb*, *Hspa5*, and *Npas2* (Fig. 4). Many of these transcripts also followed a normal circadian expression pattern, which are shown independently (Additional file 5). The smaller set of 46 transcripts upregulated by the 6^th^ hour of recovery sleep (pink cluster, Fig. 3b, Additional file 6) included transcripts with less clearly defined functions, including micro RNA and non-coding RNA and transcripts involved in RNA-binding protein sequestration (Neat1) [11].

**Fig. 5:**
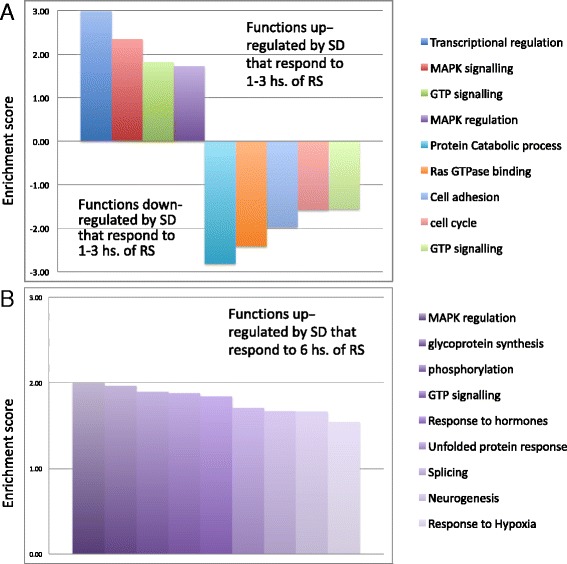
Enriched functional clusters regulated by recovery sleep. **a** Functional clusters regulated by 1–3 h of recovery sleep. **b** Functional clusters regulated by 6 h of recovery sleep. Functional annotation terms from the following databases: Gene Ontology (GO) biological process and molecular function, KEGG pathways and protein information resource keywords, were clustered based on similarity using the Database for Annotation Visualization and Integrated Discovery (see Methods). Clusters of functional terms enriched in down- or upregulated gene lists following SD as compared with the genome as a whole (*P* value <0.05) are represented as bars. Height of bars represents the enrichment score of each cluster, with the scores of downregulated clusters shown as negative numbers for visualization purposes. Enrichment score was calculated as − log(10) of the geometric mean *p*-value among all clustered terms. Only clusters with enrichment score >1.5 (average *p*-value of functional terms within the cluster <0.05) were considered. For details of the functional terms included in these clusters, as well as enriched functional terms that did not cluster with other terms, see Additional file 7

The identification of functional properties of differentially expressed genes associated with changes in sleep need is crucial for understanding mechanisms that regulate sleep and sleep function. In order to identify functional classes and pathways that respond to different amounts of recovery sleep, we performed functional annotation analysis of genes upregulated or downregulated by sleep deprivation within “fast” and “slow” responder groups. Figure 5 represents clusters of enriched functional terms among the different classes of genes (see Additional file 7 for details on clustered terms). Genes downregulated by sleep deprivation were mostly “fast” responders (*N* = 310) and were enriched in the following functional clusters: cell adhesion, protein catabolic process, Ras GTPase binding, GTP signaling and cell cycle. In addition the following terms were enriched, although they did not cluster with other similar terms: transcriptional corepression, alternative splicing, and neurogenesis (Additional file 7). For genes upregulated by sleep deprivation, “fast” responders (*N* = 52) were enriched in the following functional classes: MAPK signaling and regulation, GTPase signaling, and transcriptional regulation. The “slow” responders (*N* = 375) represented nine different enriched clusters, generally with lower enrichment scores than the upregulated fast responders despite the fact that there were substantially more genes in this group.

We also computed the decay time constant (τd) for the changes in slow and fast responding transcripts to see if they approximated the τd for the discharge of sleep need [10]. As shown in Fig. 4, these varied across functional classes. Interestingly, when we averaged τd across the different clusters of fast-responder genes the value was identical to that reported for the discharge of sleep need in this mouse strain (average τd fast responder genes: 1.3; τd for discharge of sleep need in c57/bl6: 1.3 [10]).

## Discussion

The molecular determinants of sleep homeostasis are not well known. Furthermore, transcriptional changes that track both the accumulation and discharge of sleep need have not been well characterized. Here, we present a fully integrated meta-analysis of the effects of sleep deprivation on mouse brain gene expression, by combining our data with three other previous studies [2, 3, 8]. We also provide additional evidence for genome-wide changes in cortical gene expression following various lengths of subsequent recovery sleep. We report that discovery of gene expression changes linked to either sleep deprivation or recovery sleep, and not batch effects, requires a non-traditional method of data normalization (RUV). Therefore, our study more accurately reflects true biological variance due to changes in sleep need, and vastly improves both single laboratory and meta-analysis studies of gene expression previously conducted in the absence of RUV [12].

Our results represent the first genome-wide examination of differentially expressed cortical genes that includes the response to sleep deprivation and subsequent changes across the recovery sleep period. The majority of genes fall into two general classes of transcriptional changes: transcripts that return to baseline values quickly (i.e., within the first 3 h: ‘fast responders’) and transcripts that return to baseline values slowly (by 6 h of recovery: ‘slow responders’). There was also a small subset of transcripts not affected by sleep deprivation, but upregulated in the 6^th^ hour of the recovery period. Genes that respond “fast” to recovery sleep may constitute the molecular signature underlying the discharge of sleep need based on electrophysiological studies. This is because these molecular changes parallel the discharge of sleep need as measured by changes in NREM EEG delta power. In mice, 6 h of sleep deprivation produces a large increase in delta power which then rapidly declines over the next 3 h of recovery sleep [13, 10]. This pattern is strikingly similar to the time course we find for the ‘fast’ responder genes.

The majority of fast-responders are transcripts initially *downregulated* by sleep deprivation and then upregulated with recovery sleep. Interestingly we find that these specific transcripts do not show time-of-day differences (Additional file 5) suggesting that the biological functions they serve are more closely tied to sleep homeostasis. These include genes involved in synthesis and degradation of proteins. Examples include ubiquitin-specific-proteases (Usps) and elongation initiation factors (Eifs) (Fig. 4). These results are consistent with previous studies [3, 14–16], supporting the idea that one of the immediate effects of recovery sleep is to influence protein synthesis or turnover. A second class of ‘fast’ responder genes downregulated by sleep deprivation is involved in transcriptional repression linked to histone modification. Histone acetylation and deacetylation modify the structure of chromosomes and influence access of transcription factors to DNA. We find that histone deacetylase 9 (Hdac9) and associated co-repressor Sin3A, together with the GO term “transcriptional co-repression” are downregulated by sleep deprivation (Fig. 4, Additional file 7), as previously shown in the hippocampus [8]. This suggests that part of the compensatory response is a reactivation of transcriptional repression. This may be part of the restorative function of sleep; that it re-establishes a basal level of transcription required for normal neural function. While speculative, it is possible that the cognitive deficits associated with sleep deprivation result from an unchecked expression of certain transcripts; a situation reversed during recovery sleep.

Fast responder genes *upregulated* by SD include immediate early genes previously identified as ‘induced by sleep deprivation’ (e.g. Arc, Egr1, Egr2 and Nr4a1 [2, 3, 14, 15, 17] (Fig. 4 and Additional file 5). The function of immediate early genes is primarily the regulation of transcription, a functional category that is also enriched in this group. We also find that several of the fast-responder genes are traditional ‘circadian’ genes (e.g. Arntl, Dbp, Per1, and Per2 [2]) and show time of day differences in their expression (Additional file 5). The precise role of these immediate early and clock genes in sleep homeostasis is unclear. Deletion of Arntl (BMAL1) in mice alters baseline sleep architecture, increases NREM EEG delta-power baseline conditions, and attenuates the homeostatic response to sleep deprivation [18]. In addition, Per1 and Per2 brain expression in various inbred mouse strains correlates with changes in NREM EEG delta-power [19–21], suggesting these genes are tied to the sleep homeostat. However, Per mutants have a normal response to sleep deprivation as measured by NREM delta power [22, 23], indicating they play no central role in sleep homeostasis. Similarly, the expression of the immediate early gene Homer1A also tracks sleep need [2], but Homer1A null mice have normal sleep homeostasis [24]. Therefore it is possible that the regulation of these particular clock and immediate early genes may not be as closely linked to sleep homeostasis, as appears to be the case for other clock genes [18, 20]. Instead, circadian rhythms or neural activity may play more influential roles in their expression. This interpretation is consistent with earlier studies. It has been shown that several immediate early genes, such as Per1 and Arc, are also upregulated following contextual fear conditioning or object location memory for example [15, 25, 26].

The slow responding transcripts represented the majority of all transcripts upregulated by sleep deprivation (Fig. 4). These included genes previously linked to sleep homeostasis including Homer1, Bdnf and Npas2 [27, 28, 19]. There appears to be functional overlap between these slow responding transcripts and fast responding transcripts downregulated by sleep. GO terms or functional clusters that overlap include: cell adhesion, neurogenesis, GTP signalling and splicing (Fig. 5, Additional file 7). Adhesion molecules (such as Neuroligin 1; Fig. 4, Additional file 3) are particularly interesting because they may link early responses to SD (e.g.. clock gene expression) with slower responses. This is because sleep deprivation induced changes in Neuroligin1 are dependent on clock transcription factors [29]. A much smaller subset (16 genes) was downregulated by sleep deprivation. The functions of these genes are not well understood.

Lastly, we identified a small number of genes that were unaffected by sleep deprivation but were upregulated in the 6th hour of recovery sleep (pink cluster, Fig. 3b). The function of many of these genes is also obscure. One example is the long non-coding RNA Neat1. Neat1 is retained in the nucleus where it forms the core structural component of the paraspeckle sub-organelle located within the eukaryotic nucleus [11]. Neat1 has been shown to regulate transcription via protein sequestration within paraspeckles [30]. Paraspeckles are believed to function as a reservoir of mRNA that are released into the cytoplasm under certain conditions (e.g. cellular stress) and/or provide a means of RNA sequestration. The reason for this delayed expression of transcripts is unclear. In mice, 6 h SD not only increases NREM delta power in the first three hours of recovery sleep, but also leads to a delayed ‘rebound’ in REM sleep time that can occur at or after 6 h [1]. Therefore, it is possible that the expression of this small subset of transcripts is driven by REM sleep [1, 30, 31].

While our study represents a significant improvement over previous studies that set out to identify transcripts associated with sleep homeostasis, there are some limitations. For example, our current methods do not allow for the identification of spatial resolution of specific cortical layers, or regional differences between frontal and parietal cortices. Further, the cell-type specificity for the changes in expression identified in our study is not characterized. In addition, it is difficult to differentiate which gene expression changes are responding to sleep pressure from those that are responding to stress hormones [32]. Future studies using improved techniques, such as the use of TRAP technology [33, 34] will be necessary to identify cell-type specific changes in transcripts associated with sleep homeostasis.

## Conclusions

This is the first study where RUV normalization has been used to compare multiple genome-wide data sets following sleep deprivation. We also used this approach to examine transcriptional changes during the recovery sleep period. We show that RUV vastly improves the meta-analysis of data generated in different laboratories and reveal novel changes in transcription during recovery sleep. We find that sleep produces two waves of transcription during recovery sleep. Some changes occur rapidly, others more slowly across six hours of sleep. The fast responding transcripts may represent the molecular components of sleep homeostasis as they change with a time constant that is remarkably similar to the time constant for the discharge of sleep need. Further characterization of these genes may reveal sleep function and the biological basis for sleep need.

## Methods

### Subjects

C57BL/6 J adult male mice (2 months of age) were obtained from Jackson laboratories and housed individually for a week in an experimental room on a 12 h./12 h. light/dark schedule with lights on at 7:30 am (Zeitgeber time (ZT) 0). Food and water were available ad libitum throughout the experiment. All experiments were approved by the Institution of Animal Care and Use Committee of the University of Pennsylvania and were carried out in accordance with all National Institutes of Health guidelines.

To examine gene expression in the mouse brain cortex after 5–6 h of sleep deprivation and subsequent recovery sleep we generated a dataset of 96 microarrays (see below). Groups of mice were either sleep deprived or sleep deprived and allowed recovery sleep for 1, 2, 3, or 6 h. Matching control (CC) animals were left undisturbed but sacrificed at the same time of day. Sleep deprivation was achieved by brushing the mice with a soft brush to keep them active. We did not surgically implant EEG electrodes to quantitatively measure sleep. This was done, as true for earlier studies [2, 3], to prevent changes in gene expression that might occur as a result of the surgery. However, analyses of animals implanted with EEG electrodes undergoing the same procedures shows that sleep deprivation procedures similar to ours is effective at maintaining wakefulness in a variety of mouse strains (including c57/bl6) [10, 35]. In addition, when given an opportunity to sleep, mice spend most of the subsequent 6 h period in sleep [10]. The experimental protocol was repeated daily, to obtain one animal per time point per experimental day, to gather 6–7 mice per experimental group. We also performed a meta-analysis using data from three previously published studies that tested the effect of 6 h of sleep deprivation in the mouse cortex, hippocampus and whole brain [2, 3, 8].

### Microarrays

Cortical dissections were performed by a single experimenter, and tissue was rapidly dissected and immersed in chilled RNAlater (Qiagen), kept overnight at 4 °C, then frozen at −80 °C. RNA extraction was performed using the miRNeasy kit (Qiagen). Biotinylated sense-strand cDNA were prepared from 300 ng total RNA at the UPENN molecular profiling core using the Affymetrix WT Plus Kit. Single stranded cDNA was hybridized to a Mouse Gene 2.1 ST 96-Array Plate using GeneTitan Hybridization, Wash and Stain Kit for WT Array Plates. The array plate was washed and stained in the GeneTitan multi-channel instrument. Gene 1.1 ST Array Plates were scanned using the GeneTitan® Multi-channel Instrument.

### Cross-study data integration

Data from GSE9444 [2], GSE6514 [3] and GSE50423 [8] were obtained using the R/Bioconductor package GEOquery (v. 2.36.0). Data generated in this study are publicly available through GEO (GSE78215). To allow for cross-platform comparison, Affymetrix probeset IDs were mapped to ENSEMBL gene Ids using the R/Bioconductor package biomaRt (v. 2.26.1). Probesets or ENSEMBL genes that showed multiple mappings were excluded from the cross-platform analysis only. Probesets that showed a log expression value > 4 in >50 % of the samples were included.

### Normalization and statistical analysis

We have previously shown that Removal of Unwanted Variation (RUV) [5] is a normalization method that is able to properly correct for batch effects in experimental neuroscience data obtained through RNA sequencing [4]. Any difference between biological replicates can be attributed to unwanted effects. The RUV method exploits the fact that genes that should not be changing in a biological system (negative controls) or differences between replicates, carry in their observed levels patterns of unwanted variation that can be used to adjust for unwanted effects. In this study, we used log-transformed RMA normalized data as input to RUVSeq, a Bioconductor package originally designed to perform RUV analysis on RNA sequencing data [5]. The advantage of doing so is the ease of integration with future or currently available RNA-seq studies, since RNA-seq is now the standard for the quantification of genome-wide gene expression. We compared the results of differential expression analysis of data normalized using RUV based on replicate samples with data normalized using RMA with Quantile normalization [9], the most commonly used method for microarray data. RUV using negative control samples was used to remove k factors of unwanted variation before statistical testing was performed. The choice of a proper k was determined as previously described [4], resulting in k = 5 for the meta-analysis of sleep deprivation (Fig. 1) and k = 6 for the recovery sleep analysis (Fig. 2). Differential expression analysis was performed using R/Bioconductor package limma (v. 3.26.8) comparing the sleep deprivation and recovery sleep samples to time matched circadian controls (SD vs. CC6, RS1 vs. CC7, RS2vs CC8, RS3 vs. CC8, RS6 vs. CC11). Comparisons between controls CC0, CC6 and CC1 were used to determine circadian changes in expression. Multiple testing corrections were performed using the method of Benjamini and Hochberg [36]. A cutoff of false discovery rate (FDR) <0.01 was used to assess significance. To evaluate performance, we assembled sets of independently validated positive control genes that are known to respond to 6 h of sleep deprivation or 2 h of recovery sleep (see Additional file 1 for details). RUV normalization was performed using the R/Bioconductor package RUVSeq (v. 1.0.0). Differential expression analysis was performed using R/Bioconductor package limma (v. 3.26.8).

Estimation of time constants of gene expression changes in response to recovery sleep. Non-linear regression analysis was used to quantify relationships between duration of recovery sleep and gene expression for each cluster. Non-linear least squares implemented in the R package stats (v. 3.3.0) was utilized to estimate parameters by assuming that gene expression varies according to an exponential function following sleep deprivation [10]:

$$ {\mathrm{logFC}}_{\mathrm{t}} = \mathrm{L}\mathrm{A} + \left({\mathrm{logFC}}_0\hbox{--}\ \mathrm{L}\mathrm{A}\right)\cdot {\mathrm{e}}^{\left(\hbox{-} \mathrm{t}/\uptau \mathrm{d}\right)}. $$

logFC_0_ and logFC_t_ are the mean logFC at the end of the sleep deprivation period and at time t respectively. τd is the time constant of the decreasing exponential function that approaches a lower asymptote, LA, approximated as the minimum mean logFC for each cluster. Starting τd values were estimated by visual examination of plots and optimized over subsequent iterations.

### Graphical displays

PCA plots were performed using the R/Bioconductor package EDASeq (v. 2.0.0). The heatmap was prepared using the R package gplots (v. 2.17.0) with modifications to the row dendrogram using the R package dendextend (v. 1.1.8). All other figures were generated using R base graphics and Microsoft Excel.

### Functional enrichment analysis

Affymetrix probeset ID’s were mapped to MGI symbol and ensemble gene ID’s for downstream analysis using the R/Bioconductor package mogene21sttranscriptcluster.db (v. 8.4.0). Functional annotation was based on ENSEMBL Gene IDs and performed using the database for annotation, visualization and integrated discovery v 6.7 (DAVID, https://david.ncifcrf.gov). The following functional categories were used: GO Biological Process and Molecular Function, KEGG pathways and Protein Information Resource keywords. Enrichment cutoff relative to background = EASE score <0.05. All genes present in the array were used as background for enrichment. Clustering was used to reduce complexity. Clustering parameters: similarity threshold 0.2, group membership 2.

## Additional files

Additional file 1: Positive control genes. (XLSX 44 kb)
Additional file 2: The impact of data integration on the detection of differential expression following sleep deprivation (SD). Differentially expressed genes detected, integrating all studies (Fig. 1). (XLSX 98 kb)
Additional file 3: Impact of RUV on differential expression. A) Top left. Distribution of unadjusted limma *p*-values for tests of differential expression between SD and CC samples following RMA and RUV normalization. RUV increases discovery of differentially expressed genes (genes that have a low *p*-value). Bottom. Volcano plot of differential expression (−log10 *p*-value vs log fold change) of Quantile and RUV normalized samples. Genes with and FDR <0.01 are highlighted in blue. Positive controls are circled in red. B) Top right. Distribution of unadjusted limma *p*-values for tests of differential expression between RS2 and CC samples following RMA and RUV normalization. RUV increases discovery of differentially expressed genes (genes that have a low *p*-value). Bottom. Volcano plot of differential expression (−log10 *p*-value vs log fold change) of Quantile and RUV normalized samples. Genes with and FDR <0.01 are highlighted in blue. Positive controls are circled in red. C) Bottom left. Distribution of unadjusted limma *p*-values for tests of differential expression between RS3 and CC samples following RMA and RUV normalization. RUV increases discovery of differentially expressed genes (genes that have a low *p*-value). Bottom. Volcano plot of differential expression (−log10 *p*-value vs log fold change) of Quantile and RUV normalized samples. Genes with and FDR <0.01 are highlighted in blue. Positive controls are circled in red. D) Bottom right. Distribution of unadjusted limma *p*-values for tests of differential expression between RS6 and CC samples following RMA and RUV normalization. RUV increases discovery of differentially expressed genes (genes that have a low *p*-value). Bottom. Volcano plot of differential expression (−log10 *p*-value vs log fold change) of Quantile and RUV normalized samples. Genes with and FDR <0.01 are highlighted in blue. Positive controls are circled in red. (PDF 6414 kb)
Additional file 4: Differential expression after sleep deprivation and all time point of recovery sleep following RUV normalization. (XLSX 179 kb)
Additional file 5: Differential expression due to circadian time following RUV normalization. (XLSX 58 kb)
Additional file 6: Clusters of differentially expressed genes as defined on the heatmap in Fig. 3b. (XLSX 105 kb)
Additional file 7: Details of functional annotation enrichment and clustering analysis. (XLSX 63 kb)
