# Supplementary figures and images for: Removal of unwanted variation reveals novel patterns of gene expression linked to sleep homeostasis in murine cortex
Source: BMC Genomics. 2016 Oct 25;17(Suppl 8):727. doi: 10.1186/s12864-016-3065-8 (PMC5088519; doi:10.1186/s12864-016-3065-8)

SD

RS2

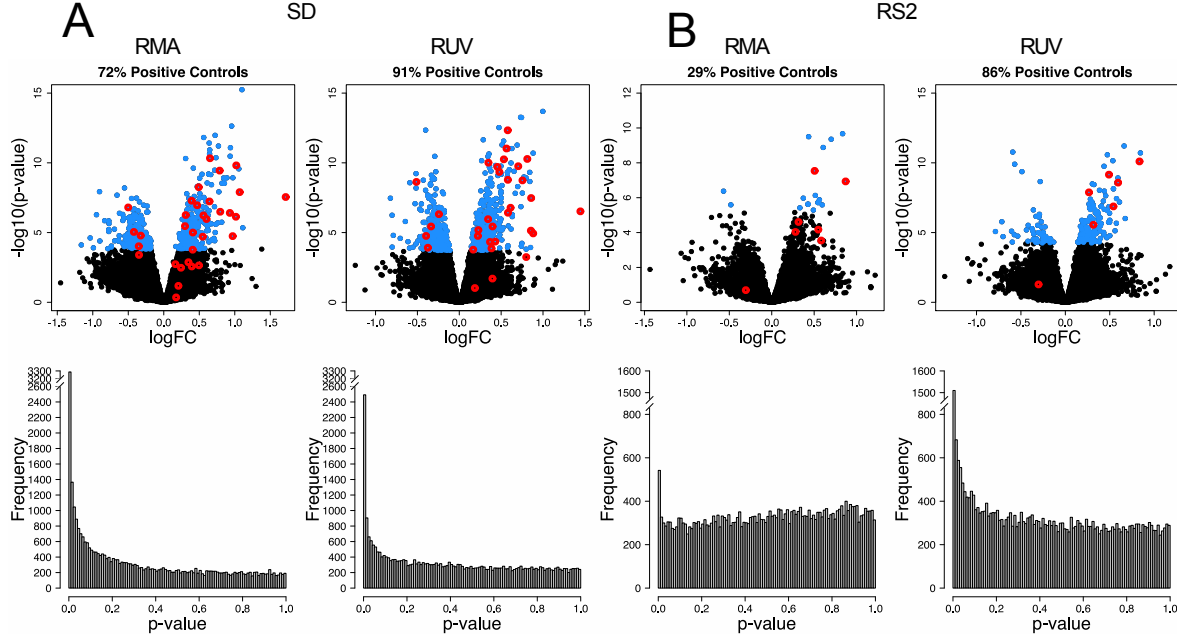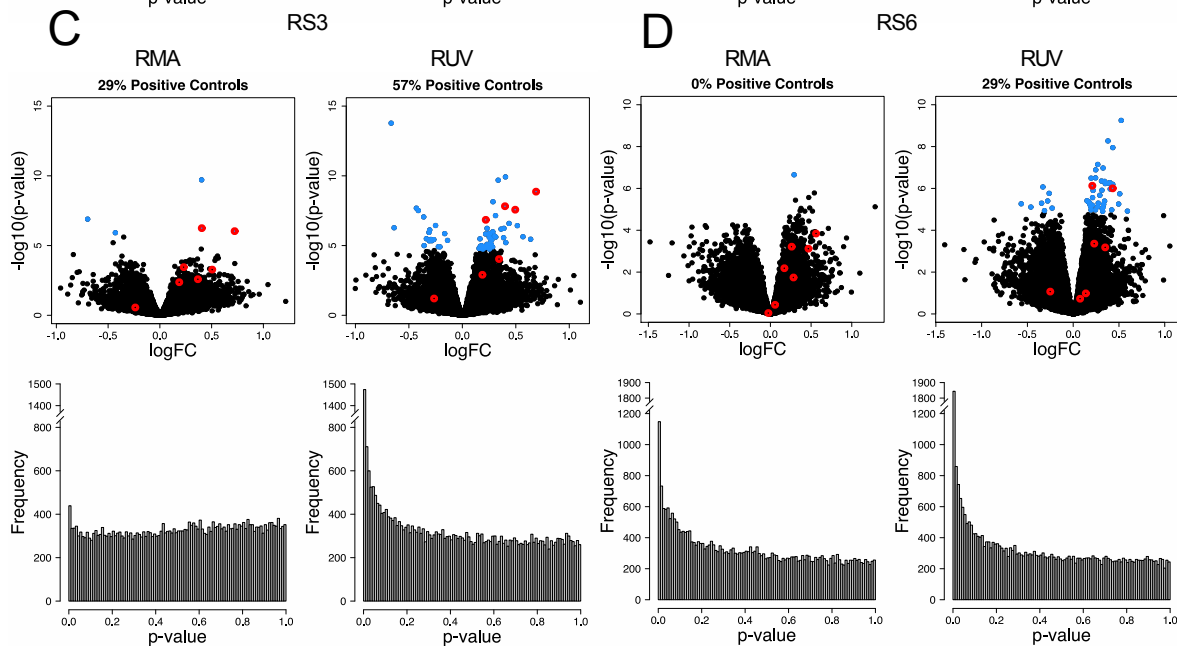

Supplement: Additional file 3: — Impact of RUV on differential expression. A) Top left. Distribution of unadjusted limma p-values for tests of differential expression between SD and CC samples following RMA and RUV normalization. RUV increases discovery of differentially expressed genes (genes that have a low p-value). Bottom. Volcano plot of differential expression (−log10 p-value vs log fold change) of Quantile and RUV normalized samples. Genes with and FDR <0.01 are highlighted in blue. Positive controls are circled in red. B) Top right. Distribution of unadjusted limma p-values for tests of differential expression between RS2 and CC samples following RMA and RUV normalization. RUV increases discovery of differentially expressed genes (genes that have a low p-value). Bottom. Volcano plot of differential expression (−log10 p-value vs log fold change) of Quantile and RUV normalized samples. Genes with and FDR <0.01 are highlighted in blue. Positive controls are circled in red. C) Bottom left. Distribution of unadjusted limma p-values for tests of differential expression between RS3 and CC samples following RMA and RUV normalization. RUV increases discovery of differentially expressed genes (genes that have a low p-value). Bottom. Volcano plot of differential expression (−log10 p-value vs log fold change) of Quantile and RUV normalized samples. Genes with and FDR <0.01 are highlighted in blue. Positive controls are circled in red. D) Bottom right. Distribution of unadjusted limma p-values for tests of differential expression between RS6 and CC samples following RMA and RUV normalization. RUV increases discovery of differentially expressed genes (genes that have a low p-value). Bottom. Volcano plot of differential expression (−log10 p-value vs log fold change) of Quantile and RUV normalized samples. Genes with and FDR <0.01 are highlighted in blue. Positive controls are circled in red. (PDF 6414 kb) [file 12864_2016_3065_MOESM3_ESM.pdf]
